# Ontogenetic color change in the tail of blue‐tailed skinks (*Plestodion elegans*)

**DOI:** 10.1002/ece3.10152

**Published:** 2023-06-06

**Authors:** Chen Yang, Siheng Chen, Jie Wang

**Affiliations:** ^1^ Sichuan Zoige Alpine Wetland Ecosystem National Observation and Research Station Southwest Minzu University Chenghu China; ^2^ Chengdu Institute of Biology Chinese Academy of Sciences Chengdu China; ^3^ School of Artificial Intelligence Beijing University of Posts and Telecommunications Beijing China

**Keywords:** chromatism, colour change, *Plestiodon elegans*, quantitatively measure, spectrometer

## Abstract

Ontogenetic color change in animals is an interesting evolution‐related phenomenon that has been studied by evolutionary biologists for decades. However, obtaining quantitative and continuous color measurements throughout the life cycle of animals is a challenge. To understand the rhythm of change in tail color and sexual dichromatism, we used a spectrometer to measure the tail color of blue‐tailed skink (*Plestiodon elegans*) from birth to sexual maturity. *Lab* color space was selected due to its simplicity, fastness, and accuracy and depends on the visual sense of the observer for measuring the tail color of skinks. A strong relationship was observed between color indexes (values of L*, a*, b*) and growth time of skink. The luminance of tail color decreased from juveniles to adults in both sexes. Moreover, we observed differences in color rhythms between the sexes, which may be influenced by different behavioral strategies used by them. This study provides continuous measurements of change in tail color in skinks from juveniles to adults and offers insights into their sex‐based differences. While this study does not provide direct evidence to explain the potential factors that drive dichromatism between the sexes of lizards, our finding could serve as a reference for future studies exploring possible mechanisms of ontogenetic color change in reptiles.

## Graphical abstract

This picture shows the whole tail color change process at different stages for *Plestiodon elegans*.

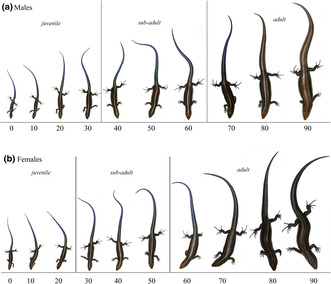

## 1 INTRODUCTION

The phenomenon of color change is commonly found in a wide range of taxonomic animal groups. The following typical color change models are prevalent: (1) slow color change (within days or months) and (2) rapid color change (within milliseconds to hours). Previous studies have focused on the mechanisms driving these models (Alfakih et al., 2022; Duarte et al., 2017; Endler, 1992; Lozano, 1994; Zahavi, 1975). The rapid color change is mostly reversible and driven by the movement towards pigment granules (dispersion or concentration), which could be used for camouflage and vigilance by animals (Endler, 1992; Ligon & McGraw, 2018). In contrast, the slow color change is mostly non‐reversible and driven by iridophores and guanine crystals arrangement associated with the normal progressive development of an individual of a species (Booth, 1990; Kuriyama et al., 2013; Zhang et al., 2022). As the regulatory mechanisms for both color change models may differ, the underlying evolutional driving forces for the color change should also differ (Stuart‐Fox & Moussalli, 2009). Relatively slow color changes and those caused by developmental plasticity are probably more common than rapid changes; however, these are less understood.

Previous studies on ontogenetic color change have focused on the head, throat, abdomen, tail, stripe, or other body parts (Hawlena et al., 2006; Sinervo et al., 2001; Smith et al., 2016). Different body regions are connected to convey messages to different receivers (Cooper et al., 1987; Martín & López, 2009; Smith et al., 2016). Compared with other body regions, many evolutionists and animal behaviorists have focused on the change in tail color owing to its diversity and specificity. *Plestiodon* spp. are often used as an important model for studying ontogenetic color change in lizards (Kuriyama et al., 2006; Richmond & Reeder, 2002). The genus *Plestiodon*—comprises 51 species that are widely distributed in the Palearctic, Oriental, and Nearctic realms (Uetz, 2023). Skinks are a diverse group of lizards that exhibit considerable variation in terms of change in tail color that is shaped by a combination of genetic, environmental, and behavioral factors (Lande, 1980; Shine, 1989). Juvenile skinks mostly possess a noticeable and vibrant blue‐colored tail that allows them to perform autotomy as a defense mechanism against predators, and subsequently, regrow their tails (Hawlena et al., 2006; Vitt & Cooper, 1986). Why do *Plestiodon* spp. adopt different tail color change patterns across species? What are the factors that drive ontogenetic changes in the tail color of *Plestiodon* spp.? Many hypotheses are applied to explain the differences in tail color among different age classes. First, the ontogenetic color change may be enforced by behavior or predation susceptibility caused by different predation pressures during the life cycle of an animal (Hawlena, 2009). Second, the intra‐species signal hypothesis suggests that the blue tail of *Plestiodon* spp. is a signal that reveals the juvenile status to reduce attracting attacks by adult conspecifics (Arnold, 1984; Fresnillo et al., 2015). Third, this hypothesis states that there is a trade‐off between tail color and size that affects the probability of predation (dos Santos Guidi et al., 2021). The fourth hypothesis suggests that the blue coloration is a signal to predators, suggesting that the lizard is noxious (Cooper & Vitt, 1985).

However, there are several limitations to the research on changes in tail color in skinks. First, a standardized, fast, and accurate method is required for color measurements. Earlier tools for color measurements included color reference charts (e.g., the Pantone color chart). A color reference chart has been widely used in various studies because it is simple, fast, and accurate for measurements (Vik, 2017). With the development of photography equipment and algorithms, it has become possible for objective measurements of color to be obtained using photography and color charts. Additionally, visual modeling can also be performed based on these measurements (van den Berg et al., 2020). It is important to note that the accuracy of color quantification can be influenced by various factors, including differences in consumer cameras, unpredictable testing conditions, and the utilization of uncalibrated photographs (Troscianko & Stevens, 2015). These challenges may significantly compromise our ability to precisely measure and compare colors, thus posing potential implications for our comprehension of the evolution of tail coloration in skinks. Typical wavelengths that can be detected by the human eye range from approximately 390 to 700 nm (visual spectrum); however, many animals can perceive a wider spectral range (i.e., ultraviolet [UV] and infrared) (Osorio & Vorobyev, 2008). To overcome this limitation, more sophisticated spectrometers with wider wavelengths (200–1200 nm) should be used for measuring color (Bybee et al., 2012; Cageao et al., 2001). Spectrometry has a major advantage in that it provides only the reflectance properties of the test target, which are not influenced by the visual system of the animal. Spectral data can be used to perform visual modeling in different color spaces depending on the research subject and question. The color perception of animals depends markedly on brightness shifts and chromatic changes (Osorio & Vorobyev, 2008). The L*a*b* color space can be utilized to establish the connection between the trichromatic visual system of species and reflectance (Fairchild, 2013; Hunt & Pointer, 2011). Second, only few studies have investigated the rhythm of slow color change (speed and magnitude of color change) over an entire life cycle. Animals endure different environmental pressures, such as food resource scarcity, predator risk, and intra‐ and interspecific competition, at different life stages (Husak et al., 2004; Ligon & McGraw, 2018; Thurman, 1988). These factors vary and are uncontrolled in the wild, and may influence ontogenetic color changes. Third, obvious differences exist in the growth rules between the sexes of skink (Yang et al., 2019); however, it is unclear how the tails of different sexes vary in terms of ontogenetic color change.

Therefore, we selected the small fade‐tail skink (blue‐tailed skink: *Plestiodon elegans*) to analyze the slow color change in its tail in the laboratory. The objectives of this study were to (1) quantitatively measure tail color using the L*a*b* color model; (2) continuously measure the change in tail color from juvenile to mature life stages; (3) compare L*a*b* values and calculate chromatism among different age groups to trace the rhythm of color change; and (4) assess the dichromatism between sexes and verify the proximate factor that drives the change in tail color. We hope that our study will serve as a reference for future research that seeks to explain the evolutionary driving forces of ontogenetic color change in the tails of skinks.

## 2 MATERIALS AND METHODS

### 2.1 Animal collection

Individuals of *P. elegans* (blue‐tailed skinks, weighing approximately 1.4 g) were obtained from a captive population in the Xiaotao Skink farm in Xing'an County, Guangxi Province, China in March 2016. Animal feeding and management, site facility, animal health and safety for the captive population were implemented in compliance with the Wildlife Husbandry and Administration Standard, China (LY/T 3214‐2020). A total of 24 skinks (13 males and 11 females) were collected 4 weeks after hatching. The lizards were placed in a plastic box filled with grass and food (insects) during transport from the farm to the laboratory at the Chengdu Institute of Biology (CIB), Chinese Academy of Sciences (CAS) (104.06° E, 30.67° N). No skink died during transportation (2 days from the sample site to the laboratory at a distance of 1100 km). All experimental procedures used in this study were approved by the Animal Care and Use Committee of the CIB, CAS. Permits for animal collection were approved by the Department of Wildlife Management, Sichuan Bureau of Forestry Administration, China. All staff, researchers, and students received appropriate training before performing the animal studies.

In our study, as the snout–vent length (SVL; tip of the head to the cloacal opening) is correlated to gonadal development, skinks were divided into three age groups based on this morphological trait (Du & Ji, 2001). We used the SVL to define age groups (juvenile, SVL <50 mm; sub‐adult, SVL <50–69 mm; adult, SVL >69 mm). We used growth curve fitting to estimate cutoff points for the three age groups. The cutoff points for male skinks were <34 weeks for juveniles, between 35 and 65 weeks for sub‐adults, and >66 weeks for adults. The cutoff points for females were <28 weeks for juveniles, between 28 and 57 weeks for sub‐adults, and >57 weeks for adults.

### 2.2 Animal husbandry

All skinks were housed in standard plastic cages (26 cm L × 17.5 cm W × 12.5 cm H) in the laboratory. A single skink was placed in each cage to avoid mutual interference and fighting. All cages were filled with a substrate of coconut soil with a thickness of 3 cm (Nomoypet Products) covered by 2–3 cm of cured hay, which provided a layer for the skinks to burrow beneath and aid in their thermoregulation. A plastic box with a diameter of 3 cm and height of 1 cm was filled with tap water and placed at the corner of the cage. Five grams of nutrient powder was added to the water each week. The powder contained vitamins A and D and various trace minerals (ReptiCalcium®, Nomoypet Products). Yellow mealworms (*Tenebrio molitor*), needle crickets (*Acheta domesticus*), and Turkistan roaches (*Blatta lateralis*) were provided as food once per week.

The experiment was performed under a 12‐h light–dark cycle condition. The light was provided by an incandescent light bulb (~70 lux) mounted on the roof from 07:00 to 19:00. Skinks may develop metabolic bone disease due to vitamin D3‐related calcium insufficiency without access to ultraviolet (UV) light (Adkins et al., 2003; Diaz et al., 2015). Thus, a UV lamp (UVB 10.0; Nomoypet Products) was installed at the top of each cage from 12:00 to 14:00 using an automatic timer. Air conditioning was used to control the ambient room temperature at 24°C. A ceramic heat lamp (100 W; ReptileStructure Products) was placed at a distance of 20 cm from each cage to maintain a constant cage temperature. Daytime thermal gradients in each cage ranged from 25 to 30°C (Shen et al., 2017).

The photoperiod was reduced to 6 h per day of light and food was withheld for the first 2 weeks to stimulate hibernation. During the subsequent 2 weeks, no light source was provided and the room temperature was kept constant at 15°C. Skinks were housed at a constant temperature of 8°C (non‐air‐conditioned room) for 8 weeks. Eight weeks later, the skinks were given food and nutrient powder and were maintained under conditions identical to the rearing conditions.

### 2.3 Color measurements

The tail color was measured using a CIELAB colorimeter, equipped with an Ava Spec‐2048 spectrometer. The spectrometer was standardized with an argon–mercury lamp (200–1200 nm). Because of perceptual uniformity, device independence, wide gamut, and easy calculation for chromatism, we chose the L*a*b* color model, which is device‐independent and based on human vision to quantify the changes in tail color. The L*a*b* model is a three‐dimensional model, where “L*” represents the lightness of the color, with 0 indicating black and 100 indicating a diffused white; “a*” represents the redness versus greenness index; and “b*” represents the yellowness versus blueness index (Fairchild, 2013). All color measurements and data were recorded using AvaSoft 8.0 software.

#### 2.3.1 Measurement of samples

We first used a standard diffused whiteboard (Aantes Inc.) to measure and save the present spectrum in a light and dark environment as a reference. The integration time for contact sample measurement was 24.5 ms. The integrated area was 3 mm^2^. The test conditions for the measured geometry (45°), angle of view (2°), and illuminant (*D*_50_) were standardized to reduce error in each test. Then, we captured the spectral reflectance (%) of each skink's tail using a reflection probe placed in the middle section from the vent to the tail end of a whole tail. Each skink was measured twice and the mean value was used for the study. Each individual was measured once per week, and data collection was not conducted during weeks 37–48 due to hibernation.

#### 2.3.2 Spectral data processing

To obtain the L*a*b* value from the spectral reflectance, two color space conversions were required. The first step was to convert spectral reflectance to the *XYZ* color space, then convert the *XYZ* value to the L*a*b* value (Yang et al., 2021).

#### 2.3.3 Estimation of chromatism

Four indexes were used to describe color change, namely ΔL*, Δa*, Δb*, and ΔE*, to estimate chromatism, where *n* represents the week after a skink hatched and *n* − 1 represents the week before that; ΔL* is the difference in luminance indicated by the difference in L* value between two measurements; Δa* is the chromaticity difference indicated by the difference in a* value between two measurements; Δb* is the chromaticity difference indicated by the difference in b* value between two measurements; and ΔE* is calculated based on the difference between the chromaticity (a* and b*) and lightness (L*) using the Euclidian distance in the following equation. The values of $L_{1}^{*},a_{1}^{*},b_{1}^{*},△L_{1}^{*}△a_{1}^{*},△b_{1}^{*},\text{and}△E_{1}^{*}$ were measured in the juveniles. The values of $L_{2}^{*},a_{2}^{*},b_{2}^{*},△L_{2}^{*}△a_{2}^{*},△b_{2}^{*},\text{and}△E_{2}^{*}$ were measured in the sub‐adults. The values of $L_{3}^{*},a_{3}^{*},b_{3}^{*},△L_{3}^{*}△a_{3}^{*},△b_{3}^{*},\text{and}△E_{3}^{*}$ were measured in the adults.

$$△L^{*}=L_{n}^{*}-L_{n-1}^{*}$$

$$△a^{*}=a_{n}^{*}-a_{n-1}^{*}$$

$$△b^{*}=b_{n}^{*}-b_{n-1}^{*}$$

$$△E^{*}=\sqrt{\left(△{L^{*}}^{2}+△a^{*2}+△b^{*2}\right)}$$

### 2.4 Statistical analysis

All continuous variables were tested for normality using the Kolmogorov–Smirnov test and transformed variables were tested when needed to meet the assumptions of least‐squares regression and generalized linear models (GLM). Because each skink was measured twice, we used the intraclass correlation coefficient (ICC) to evaluate the reliability between two color measurements and estimate the difference between the two tests. Two‐way random model and consistency type were considered in the ICC analysis, which revealed a higher agreement between two independent tests. The average of two tests was used for subsequent data analysis. Because the same skink was measured in different age groups, the GLM *repeated measures* procedure was used to analyze variance when the same individual was measured several times. To compare the difference in L*a*b* values among three different age groups, we set L*a*b* values as dependent variables, sex as the between‐subjects variable with two levels (male and female skinks) and time as the within‐subjects variable with three levels (juveniles, sub‐adults, and adults). The interaction term (sex × time) was selected to determine if sex groups changed over time in the same pattern. If the interaction term was significant (*p* < .05), it indicated that both sexes changed in different ways. Tukey's honestly significant difference test was conducted for post‐hoc comparisons between all combinations of any two age groups. Thereafter, a two‐tailed independent‐samples *t‐*test was conducted to compare the color indexes (L*a*b* values) and chromatism index (△L*, △a*,△b*, and △E*) between sexes. Additionally, linear regression analyses between color index (L*a*b* values) and age were performed for both male and female skinks. To examine variation in color change with growth time, we calculated regression slopes using ordinary least‐squares regression. Pearson's correlation was used to examine the relationships between color index (L*a*b* values) and age. All results were considered significant if *p* < .05. All statistical analyses were performed using SPSS 19.0 (IBM).

## 3 RESULTS

### 3.1 Color change among age groups

**FIGURE 1:**
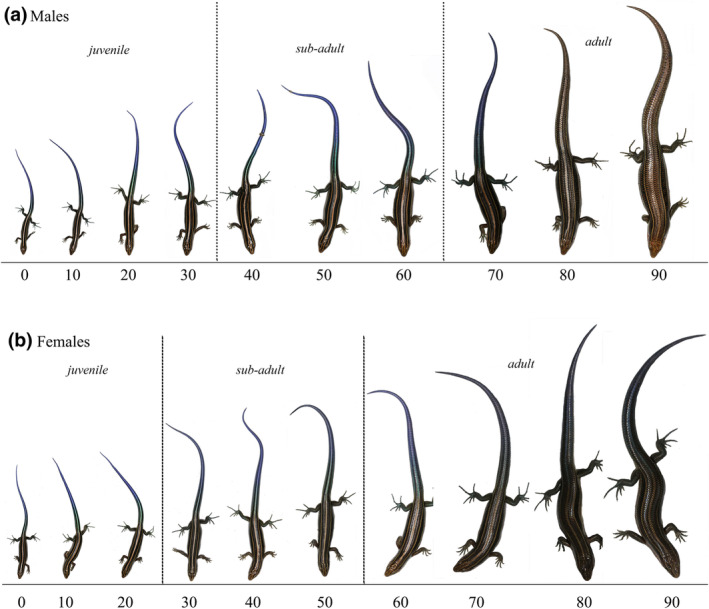
Tail color change at different stages of male (a) and female (b) *Plestiodon elegans*. Numbers in the horizontal axis are weeks after birth.

**FIGURE 2:**
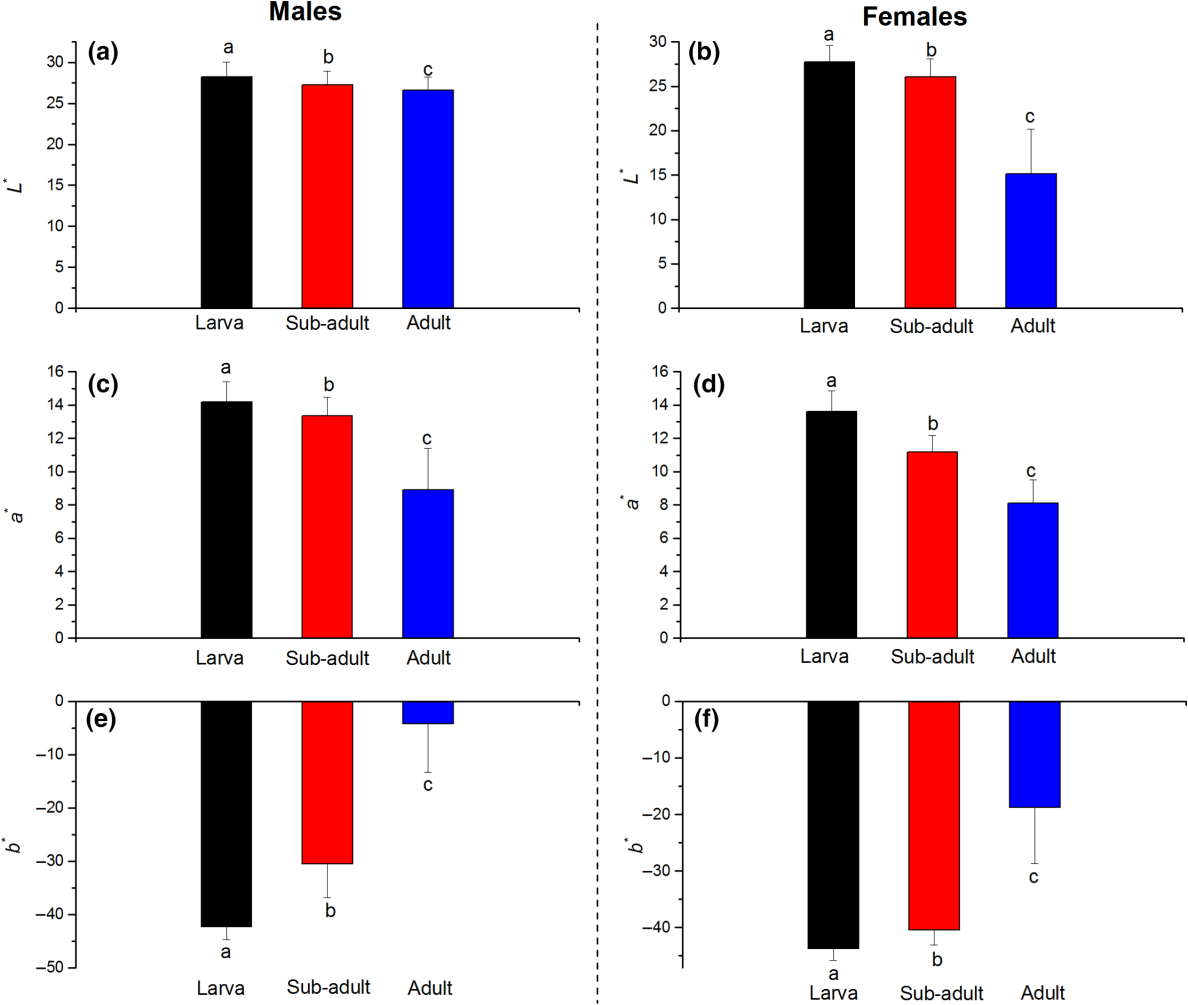
Distributions of tail colors (L*a*b* values) for the *Plestiodon elegans* population at three age groups. (a) L* for male skink; (c) a* for male skink; (e) b* for male skink; (b) L* for female skink; (d) a* for female skink; (f) b* for female skink. Error bars are +SD. Different letters indicate statistically significantly different across age groups (GLM Repeated Measures, *p* < .05) according to *Tukey's* honestly significant difference tests.

**FIGURE 3:**
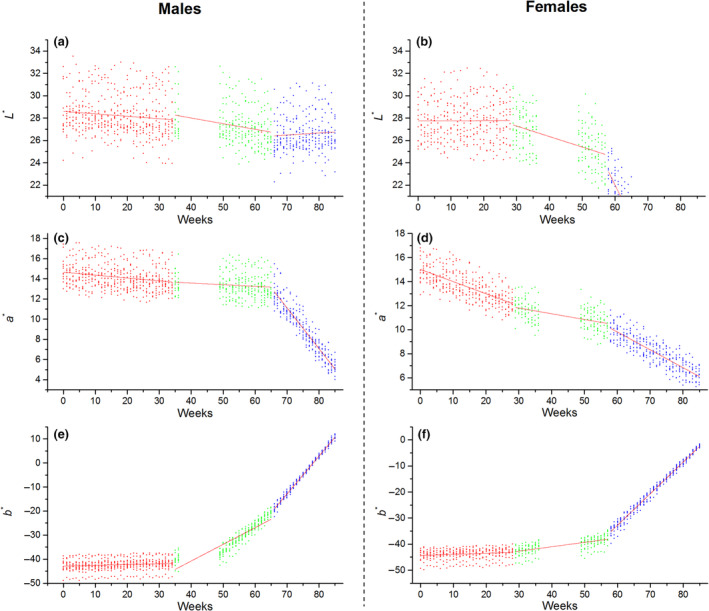
Relationship between ages and tail color variables measured in two gender groups. Lines were fitted with generalized model. Male juvenile was <34 weeks (red color); sub‐adult male is from 35 to 65 weeks (green color); adult male is more than 66 weeks (blue color). Female juvenile was <28 weeks; sub‐adult female is from 28 to 57 weeks; adult female is more than 57 weeks. Break period is from 37 to 48 weeks (hibernation).

The blue tail color faded in both sexes and varied with ontogenetic development (Figure 1). This was evident from the quantitative spectrophotometric analysis that revealed shifts in a* and b* values (Table 1). The within‐subjects test indicated that there is a significant time effect, in other words, significant differences were found in L*a*b* among different age groups (GLM Repeated Measures; L*: *F*_3,2_ = 2109.85, a*: *F*_3,2_ = 2554.39, b*: *F*_3,2_ = 5743.67; *p* < .01). The juvenile tail had the highest values for a* and L*, and the lowest value for b* after hatching, resulting in a bright blue color in the tail of the hatched skink. The L*and a* values slightly decreased in the tail of the sub‐adult, whereas the b* value significantly increased. The tail color of the sub‐adult skink changed to dark blue (Figure 2). The tail of the sexually mature adult skink had the lowest values for a* and L*, and the highest value for b*. The blue color of the tail of the adult skink faded and changed to brown and to dark yellow (Figure 3).

**TABLE 1:** Differences in color change among three age groups between the sexes using independent‐sample *T* test. The average value plus or minus standard deviation is presented for each sex and variable within each of the three age groups.

Groups	Variables	Sexes		Statistic test	
		Males (*n* = 13)	Females (*n* = 11)	*t*	*p*
Larva	$L_{1}^{*}$	28.23 ± 1.84	27.71 ± 1.86	3.43	<.01
	$a_{1}^{*}$	14.17 ± 1.24	13.60 ± 1.25	6.35	<.01
	$b_{1}^{*}$	−42.31 ± 2.38	−43.74 ± 2.05	8.71	<.01
	$△L_{1}^{*}$	−0.02 ± 1.054	0 ± 1.03	−0.29	.77
	$△a_{1}^{*}$	−0.03 ± 0.82	−0.09 ± 0.82	0.89	.37
	$△b_{1}^{*}$	0.03 ± 0.77	0.03 ± 0.79	−0.01	.99
	$△E_{1}^{*}$	1.40 ± 0.65	1.39 ± 0.64	0.18	.86
Sub‐adult	$L_{2}^{*}$	27.26 ± 1.71	26.03 ± 1.83	6.36	<.01
	$a_{2}^{*}$	13.35 ± 1.12	11.17 ± 0.99	26.48	<.01
	$b_{2}^{*}$	−30.52 ± 6.32	−40.42 ± 2.70	−11.15	<.01
	$△L_{2}^{*}$	−0.06 ± 1.08	−0.24 ± 1.17	1.71	.09
	$△a_{2}^{*}$	−0.04 ± 0.87	−0.10 ± 0.83	0.79	.43
	$△b_{2}^{*}$	1.13 ± 0.81	0.40 ± 0.87	9.03	<.01
	$△E_{2}^{*}$	1.83 ± 0.70	1.62 ± 0.64	3.25	<.01
Adult	$L_{3}^{*}$	26.59 ± 1.63	15.15 ± 5.00	37.85	<.01
	$a_{3}^{*}$	8.89 ± 2.50	8.11 ± 1.40	4.67	<1
	$b_{3}^{*}$	−4.20 ± 9.12	−18.80 ± 9.87	18.19	<.01
	$△L_{3}^{*}$	−0.02 ± 1.20	−0.61 ± 1.20	5.87	<.01
	$△a_{3}^{*}$	−0.38 ± 0.83	−0.15 ± 0.84	−3.27	<.05
	$△b_{3}^{*}$	1.56 ± 0.85	1.22 ± 0.82	4.85	<.01
	$△E_{3}^{*}$	2.18 ± 0.81	2.00 ± 0.82	2.63	<.05

### 3.2 Color change between sexes

The between‐groups test indicates that the variation between the sexes is significant (GLM Repeated Measures; L*: *F*_3,2_ = 381.90, a*: *F*_3,2_ = 56.78, b*: *F*_3,2_ = 610.90; *p* < .01). The interactions of sex × ages were significant (L*: *F*_3,2_ = 1108.48, a*: *F*_3,2_ = 121.40, b*: *F*_3,2_ = 624.31; *p* < .01), which indicates that the changes in color varied among the sexes and age groups in different ways. The sexual difference in tail color (L*a*b* values) was first recognized in the juvenile period. In fact, a minor difference was found between the sexes in the juvenile period (differences for *L*_1_*, *a*_1_*, and *b*_1_* were 0.52, 0.57, and 1.43, respectively) (Figure 2a,b). Furthermore, the coefficients of the slope for L*, a*, and b* were <0.1 during the juvenile period, indicating a slow change in color, with the tail remaining bright blue in the juvenile period as predicted (Table 1). In the sub‐adult period, the divergence in tail color was fast (differences $L_{2}^{*},a_{2}^{*},\text{and}\;b_{2}^{*}$ were 1.23, 2.18, and 9.90, respectively) (Figure 2c,d). Furthermore, for both sexes, slight decreases in L* (male: slope = −0.050 vs. female: slope = −0.094) (Figure 3a,b) and a* (male: slope = −0.016 vs. female: slope = −0.048; Figure 3c,d) were recorded. However, the value for b* for males (27.71 ± 1.86) had a greater increase compared with that for females (−40.42 ± 2.70), indicating that the tail color of males faded to a deeper yellow color compared with that of females (independent‐samples *t‐*test: *t* = −11.15, *p* < .01; Table 2). After sexual maturity, the sexual difference in terms of L*a*b* continued to increase (differences for $L_{3}^{*},a_{3}^{*},\text{and}\;b_{3}^{*}$ were 11.14, 0.78, and 14.60, respectively) (Figure 2e,f). The chromatic values (a* and b*) continued to change with growth time in both sexes (all *R* > .85, *p* < .05; Table 1). The luminance for female skinks continuously decreased (slope = −0.597, *p* < .01), whereas the luminance for male skinks remained stable (slope = 0.019, *p* > .05; Table 2).

**TABLE 2:** Summary results of linear regression between age and color change (L*a*b* values) for both sexes. Weeks is set as the independent variable, and color index (L*a*b* values) are dependent variable.

Age groups	Variables	Males					Females				
		Sample size (*n*)	Intercept	Slope	*R*	*p*	Sample size (*n*)	Intercept	Slope	*R*	*p*
Larva	$L_{1}^{*}$	455	28.60	−0.02	−0.12	<.01	319	27.76	0.00	.00	.98
	$a_{1}^{*}$	455	14.65	−0.03	−0.23	<.01	319	15.01	−0.10	−.68	<.01
	$b_{1}^{*}$	455	−42.89	0.04	0.15	<.01	319	−44.34	0.04	.17	<.01
Sub‐adult	$L_{2}^{*}$	247	30.03	−0.05	−0.24	<.01	187	30.11	−0.09	−.49	<.01
	$a_{2}^{*}$	247	14.22	−0.02	−0.11	.09	187	13.23	−0.05	−.51	<.01
	$b_{2}^{*}$	247	−68.48	0.69	0.89	<.01	187	−47.74	0.17	.66	<.01
Adult	$L_{3}^{*}$	260	25.19	0.02	0.06	.29	308	57.88	−0.60	−.97	<.01
	$a_{3}^{*}$	260	39.39	−0.40	−0.94	<.01	308	18.71	−0.15	−.86	<.01
	$b_{3}^{*}$	260	−122.71	1.57	0.99	<.01	308	−105.47	1.21	.99	<.01

## 4 DISCUSSION

The color of the tail of blue‐tailed skink remains bright blue before sexual maturation and fades to brown or a dark color within several days or months following sexual maturation (Figure 1). The color change in the skink tail is a physiological change induced by a special type of cells called iridophores in the dermis, as recent research has shown that guanine crystals that are arranged in a specific manner create blue color (Zhang et al., 2022). Change in tail color was found to be nonlinear among different age groups and may be correlated to environmental pressures in different growth periods (Figure 3). Tail color may play different roles in different age groups.

The quantitative measurement of tail color in two dimensions (luminance and chrominance) shows that both female and male juvenile skinks had a bright blue tail, with high L* and a* and low b* (Table 1; Figure 2a,b). Previous research on *Eumeces* spp. found that ontogenetic color change in the tail is congruent with behavioral change (Blanckenhorn, 2005; Clark & Hall, 1970). A bright blue tail is often used as a decoy for predator avoidance and juvenile skink forage in higher‐risk environments where it may be the most needed (Hawlena et al., 2006; Vitt & Cooper, 1986). Controlled experiments confirmed that the blue tail is an effective decoy for attracting attacks toward a non‐vital body part (Watson et al., 2012). Our results based on L*a*b* values indicated that the tails of juveniles are quantifiably brighter and bluer than those of other age groups, which may be used to test related hypotheses.

Previous research on ontogenetic color change in lizards mostly focused on juveniles and adults (Belliure et al., 2018; Tong et al., 2019). Color change in sub‐adults was less studied even though both sexes need to build a competitive advantage in terms of breeding. Sub‐adults of *P. elegans* show more rapid growth morphology (SVL, tail length, and head size) compared with juveniles (Du & Ji, 2001; Yang et al., 2019). Compared with the juvenile period, the chromatic values of the tail (a* and b*) changed in the sub‐adult period, with luminance (L*) retaining a high value (Figures 2 and 3). Owing to the rapid morphological growth, sub‐adult skinks are more explorative and aggressive (Thompson, 2021). Increase in exploration and aggression enables individuals to cover a larger home territory, leading to increased energy intake and potential for finding a mate to achieve success in reproduction. However, being more explorative and aggressive also implies a high risk of encounters with predators and an increase in injury (Coladonato et al., 2020; De Meester et al., 2022). Our data suggest that individuals in the sub‐adult period are selected to retain high luminance and low chrominance (Table 1). This finding can support related research on coloration change linked to behavioral strategy (Hawlena et al., 2006; Nasri et al., 2018).

The tail color of both sexes is visually different during the final sexual maturity period (Figures 1 and 2). The blue color of the tails of sexually mature adults of both sexes faded and the L*a*b* values markedly shifted and had steeper slopes compared with the other two age classes (Figure 3). The adult tail color was found to change in a short duration after the maximum morphological trait (SVL) was achieved (Figure S1). As the tail area increases with body size, it may lead to higher detectability by major predators such as birds or mammals (dos Santos Guidi et al., 2021). Tail color patterns are identified as defense strategies, a brown and black tail color is better for camouflage with the background (soil, leaves, etc.) compared with that offered by a blue tail color, and should further reduce detectability by predators (Fleishman & Persons, 2001). Moreover, the efficacy of conspicuous tails as a decoy may rely on associated behavioral displays. We speculate that male skinks spend more time moving for energy intake and search for a mate, and hence a bright tail could be a lure to reduce predation risk (Clark & Hall, 1970). In contrast to male skinks, female skinks having a dark tail are considered a conservation tactic. As a darker tail offers better matching with the background, adult female skinks develop longer and heavier tails, which are beneficial for energy investment (Scharf & Meiri, 2013; Stuart‐Fox & Moussalli, 2009).

A spectrometer was used to quantitatively measure color using L*a*b* values and a connection was established between color and morphological traits. Blue tails may benefit both male and female juvenile and sub‐adult skinks by reducing deadly predator attacks during forage in high‐risk environments (Figures 2 and 3). Furthermore, dichromatism may be enforced by the different behavior strategies between the sexes. The male adult skink may risk higher predator detection rates for brighter luminance to search for a mate. However, adult females may prefer a dark tail with lower predator detection rates and conserve energy for reproduction. Additionally, the change in tail color was a common phenomenon in lizards, the patterns of color signal and change differed across species (Murali et al., 2018). Therefore, future studies should investigate the correlation between behavioral strategy and color change between the sexes and explore the effect of morphological development and energy allocation on color change to better understand the evolutionary origins of the blue tail in lizards. Control experiments are also needed to fully understand whether the change in the tail color in skinks at different life stages is due to natural or sexual selections or both.

## 5 CONCLUSIONS

In this study, a spectrometer and the L*a*b* color model were used to continuously measure the change in the tail color of *P. elegans*. The changes in the tail color of *P. elegans* significantly differed among the three age groups, which may be associated with trade‐offs in energy savings to either growth or predator avoidance. Changes in tail color are dichromatic and may be enforced by different behavioral strategies between the sexes after maturity. However, caution must be exercised when explaining the mechanism underlying the ontogenetic color change owing to a shortage of comparative analysis among multiple species.

## AUTHOR CONTRIBUTIONS

**Chen Yang:** Conceptualization (equal); writing – original draft (equal). **Siheng Chen:** Data curation (equal); investigation (equal); visualization (equal). **Jie Wang:** Conceptualization (equal); methodology (equal); writing – original draft (equal).

## CONFLICT OF INTEREST STATEMENT

The author declares no conflict of interest.

## Supporting information


Figure S1.

## Data Availability

All the data generated for this study have been uploaded to FigShare: https://doi.org/10.6084/m9.figshare.21521343.v3.
