# Supplementary material for: Ontogenetic color change in the tail of blue‐tailed skinks (Plestodion elegans)
Source: Ecol Evol. 2023 Jun 6;13(6):e10152. doi: 10.1002/ece3.10152 (PMC10242887; doi:10.1002/ece3.10152)
Supplement: Supplementary file 1 — Figure S1. [file ECE3-13-e10152-s001.docx]

# Additional File

**

**

**Fig. S1** Box plots illustrating the value of chromatism (△*L*^*^, △*a*^*^, △*b*^*^, △*E*^*^) among the three age-groups. The horizontal lines across are median. (a): △*L^*^* for male skink; (c): △*a^*^* for male skink; (e): △*b^*^* for male skink; (g): △*E^*^* for male skink; (b): △*L^*^* for female skink; (d): △*a^*^* for female skink; (f)**:** △*b^*^* for female skink; (h): △*E^*^* for female skink; The bottom and top limit of each box are the lower and upper quartiles (25% and 75%, respectively). Different letters above the box indicate significant differences among age-groups. Details for calculating are in methods materials.
